# Transfer Assessment of Carbendazim Residues from Rape Flowers to Apicultural Products

**DOI:** 10.1155/2017/6075405

**Published:** 2017-01-26

**Authors:** Ying-Hong Li, Bei-Lei Zhou, Ming-Rong Qian, Qiang Wang, Hu Zhang

**Affiliations:** ^1^Zhejiang Institute for Food and Drug Control, Hangzhou 310052, China; ^2^Institute of Quality and Standard for Agricultural Products, Zhejiang Academy of Agricultural Sciences, Hangzhou 310021, China

## Abstract

Carbendazim is usually used to control the* Sclerotinia sclerotiorum* of rapes during the flowering period. This paper presents a study on transfer assessment of carbendazim residues from rape flowers to apicultural products. In the field trials, the rapes were sprayed with carbendazim on standard dosage. Bees produced apicultural products (bee pollen, honey, and royal jelly) from sprayed rapes. Apicultural products were collected on a regular basis. Carbendazim residues were extracted from bee pollen, honey, and royal jelly, respectively. HPLC/ESI-MS/MS method was developed and partially validated to identify and quantify carbendazim residues. The limits of quantification in pollen, honey, and royal jelly were 0.01 mg/kg. Mathematical curve fitting was carried out on the basis of transfer assessment of carbendazim residues from rape flowers to apicultural products. The respective carbendazim residues were 1.10 ± 0.03 mg/kg in pollen on 18th day, 0.032 ± 0.001 mg/kg in honey on 24th day, and 0.077 ± 0.002 mg/kg in royal jelly on 22nd day. Transfer assessment and mathematical curve fitting of carbendazim residues from rape flowers to apicultural products show carbendazim diminished over spraying time. The gap of carbendazim residues between pollen and honey is decreased with time. The carbendazim residues in pollen are 10 times higher than that of honey and jelly.

## 1. Introduction

Pesticides have played an important role in controlling, seducing, and preventing pests and, hence, indirectly increasing food production. The use of pesticides in agricultural activities has adverse effects towards human, which can be detrimental to food safety. Many studies have focused on the direct pollution on food from residual pesticides. Moreover, some reports suggested that pesticide residues could transfer to animal food through animal feeds [1–3]. But little research has been performed on pesticide residues in apicultural matrixes from field samples.

Apicultural products such as bee pollen, honey, and royal jelly are popular agricultural products in the world. Apicultural products have been found to exhibit interesting bioactivities, such as antimicrobial, anti-inflammatory, and antioxidant activities [4]. In China, annual output and exports of apicultural products are always ranked first in the world. Nectar plants are the source and basis for bees to collect nectar and pollen. Rapes are the main nectar plants for bees in terms of sown area in China. Rapes are widely cultivated in the Yangtze River Basin of China due to wide application and high economic value.

However, rapes easily suffered from diseases damage during whole growth period, which are significant factors threatening the rapeseed yield [5]. In agricultural practice for rape fields, fungicides as certain category of pesticides are always used for protecting rapes [6]. Carbendazim is one of the benzimidazole fungicides, which has good control effects against* Sclerotinia sclerotiorum* of rapes in the rape flowering. However, rape flowers are polluted due to spraying the pesticides during rape flowering period and will lead to pesticide residues in apicultural products. For example, pollen of oilseed rape was heavily contaminated with a broad range of pesticides, as was the pollen of wild flowers growing nearby. Consequently, pollen collected by both bee species also contained a wide range of pesticides, notably including the fungicides carbendazim, boscalid, flusilazole, metconazole, tebuconazole, trifloxystrobin, neonicotinoids, thiamethoxam, thiacloprid, and imidacloprid [7]. Therefore, level of pesticide residues in apicultural products is closely related to the pollution degree of rape flowers. While the inconvenient truth is that exports of Chinese apicultural products are blocked by technical barriers to trade due to the abuse of chemical pesticides [8], European Union and Japan have strengthened controls on pesticide residue in apicultural products. Therefore, it has great significance to detect pesticide residues in apicultural products. Many researches are carried out to determine the pesticide residues in final apicultural products. LC-MS/MS and GC-MS/MS for the determination of 200 pesticides and pesticide metabolites in honeybee samples have been developed and validated [9]. Especially to deserve to be mentioned, there are studies on pesticide residue transfer rates or percent, such as transfer assessment of fipronil residues from feed to cow milk [1] and pesticide residue transfer rates (percent) from dried tea leaves to brewed tea [10, 11]. However, the domestic researches on pesticide accumulation from nectar plants to apicultural products are rare [12, 13]. Honey bee (*Apis mellifera carnica*) colonies were placed in two apple orchards treated with the insecticides diazinon and thiacloprid and the fungicide difenoconazole in accordance with a Protection Treatment Plan in the spring of 2007, and pollen and bee bread were collected from combs inside the hives to determine the residues of pesticides [14]. Concentration levels of 30 pesticide residues were measured in honey samples collected from apiaries in northern part of Poland (Pomerania) using method based on the QuEChERS Extraction followed by HPLC/ESI–MS/MS, and 29% of the samples were found positive for at least some of the target compounds [15]. It shows the necessity and urgency to carry out relevant research.

In the current study, the field trials were designed to spray carbendazim over rapes during the flowering period. Apicultural products (bee pollen, honey, and royal jelly) obtained by bees from sprayed rapes were collected on a regular basis. The transfer assessment of carbendazim residues from rape flowers to apicultural products was figured out by HPLC/ESI-MS/MS method. Mathematical analysis for curve fitting of transferring pattern was identified by computer associated calculation. This study aimed to improve security level of apicultural products and provide basis data to establish maximum residue limits (MRL) and safe intervals of corresponding fungicides.

## 2. Materials and Methods

### 2.1. Materials and Chemicals

Methanol and acetonitrile with LC-MS grade were purchased from Merck (Darmstadt, Germany). Acetic acid (≥99.7% purity) was obtained from Sigma-Aldrich (St. Louis, USA). The Oasis HLB cartridges (6 cc/150 mg, 30 *μ*m) were provided by Waters Ltd. (USA). QuEChERS Extraction kits (1.5 g anhydrous sodium acetate and 6 g anhydrous MgSO_4_, 50 mL centrifuge tubes) and QuEChERS Clean-Up kits (150 mg anhydrous MgSO_4_, 50 mg Primary and Secondary Amine (PSA), and 50 mg C_18_, 2 mL centrifuge tubes) were purchased from Phenomenex Inc. (USA). Water (18.2 MΩ·Cm) used for reagent and sample preparation was from a Barnstead Nanopure system (Thermo Scientific, USA). All other chemicals were of analytical reagent grade. Analytical standards of carbendazim (purity 99.0%) and the internal standard carbendazim-D3 (purity 99.0%) were purchased from Dr. Ehrenstorfer GmbH (Augsburg, Germany). Stock standards (approximately 100 mg/L) of the individual compounds were prepared by dissolving the reference compounds in methanol. Working standards at lower concentrations were prepared by serial dilution of the stock standards by blank matrixes.

### 2.2. Field Trials

Field experiments were designed scientifically, in accordance with the field trial standard of pesticide residue. The field experiments were conducted during March-April 2014. The trials were conducted in experimental fields located in Zhejiang academy of agricultural sciences (Hangzhou, China). Zheshuang 72 double low Brassica, as a typical Brassica napus and breed by Zhejiang academy of agricultural sciences, was registered and released by national variety certification in 2001. Zheshuang 72 was chosen as the experiment rape. During the experiment, total 13816 plants of Zheshuang 72 were transplanted averagely to four GP 622 single-span greenhouses with the organic farming system. Each single-span greenhouse was constructed by high-strength stainless steel wire mesh. The greenhouse was about 288 m^2^, 48 meters in length, 6 meters in width, and 3.1 meters in height. Four GP 622 single-span greenhouses were numbered from P_1_ to P_4_. P_1_ greenhouse was set as blank control plot. P_2_ to P_4_ greenhouses were set as paralleled experimental plots. All rapes planted in P_2_ to P_4_ greenhouses were treated with 50% carbendazim WP (Pianjing plant protection Technology Co., Ltd, Suzhou, China) at flowering period by Jecto HD 400 hand-operated knapsack sprayer. The spraying concentration of pesticide was 432 mg/kg as recommended max-concentration (1500 g.a.i./ha). After the pesticide spraying, 4 beehives about 5000 bees in each hive were placed in P_1_ to P_4_ greenhouses, respectively. Apicultural products (bee pollen, honey, and royal jelly) from P_1_ to P_4_ green houses were collected on a regular basis as shown in Table 1. All apicultural products were carefully labeled and immediately transported and stored in the laboratory at −20°C until analyzed.

### 2.3. Sample Preparation and Extraction Procedure

#### 2.3.1. Bee Pollens

Sample extraction and clean-up procedures followed the buffered QuEChERS (Quick, Easy, Cheap, Effective, Rugged, and Safe) method [16–18]. The homogenized pollen (3 g) was weighted into a 50 mL centrifuge tube, followed by addition of 0.3 mL 1.0 mg/L carbendazim-D3 internal standard working solution. 12 mL purified water and 15 mL acetonitrile with 1% acetic acid were added after staying in static state for 20 min. Then the tube was shaken vigorously for 1 min using a vortex mixer. QuEChERS Extraction kits with 6 g anhydrous MgSO_4_ and 1.5 g anhydrous sodium acetate were added. Then the tube was shaken vigorously for 5 min using a vortex mixer and centrifuged at 5,000 rpm for 5 min. 1 mL of the acetonitrile extracts (upper layer) was transferred into a 2 mL centrifuge tube named as QuEChERS Clean-Up kits with 50 mg PSA, 50 mg C_18_, and 150 mg anhydrous MgSO_4_. After shaking and centrifugation, 0.5 mL of the upper layer was transferred into a 2 mL centrifuge tube containing 0.5 mL purified water. The vial was capped and then vortexed for 30 s, and the final solution was filtered through a 0.22 *μ*m filter for HPLC/ESI-MS/MS analysis.

#### 2.3.2. Bee Honey

The homogenized honey (5 g) was weighted into a 50 mL centrifuge tube, followed by addition of 0.5 mL 1.0 mg/L carbendazim-D3 internal standard working solution; 25 mL purified water was added after staying in static state for 20 min. Then the tube was shaken vigorously for 1 min using a vortex mixer and centrifuged at 5,000 rpm for 5 min. The upper layer solution was transferred at 5 mL/min into the Oasis HLB cartridge (6 cc/150 mg, 30 *μ*m), which was conditioned previously with 5 mL of methanol and 5 mL of water. The column was then washed with 15 mL of water for cleaning up. 10 mL methanol was used for eluting and the elution liquid was collected. The elution liquid was immersed in a gentle stream of nitrogen and concentrated to dryness. Finally 5 mL of 50% methanol solution was added to restore the volume. The final solution was filtered by 0.22-*μ*m membrane and then determined by HPLC/ESI-MS/MS.

#### 2.3.3. Bee Jelly

The homogenized jelly (2 g) was weighted into a 50 mL centrifuge tube, followed by addition of 0.1 mL 1.0 mg/L carbendazim-D3 internal standard working solution; 10 mL purified water was added after staying in static state for 20 min. Then the tube was shaken vigorously for 1 min using a vortex mixer and then volume to 20 mL by addition of methanol. Then the tube was shaken vigorously for 1 min again and centrifuged at 5,000 rpm for 5 min. 10 mL of upper layer solution was diluted by 25 mL water and transferred at 5 mL/min into the Oasis HLB cartridge (6 cc/150 mg, 30 *μ*m) after mixing, which was conditioned previously with 5 mL of methanol and 5 mL of water. The column was then washed with 15 mL of water for cleaning up. 10 mL methanol was used for eluting and the elution liquid was collected. The elution liquid was immersed in a gentle stream of nitrogen and concentrated to dryness. Finally 5 mL of 50% methanol solution was added to restore the volume. The final solution was filtered by 0.22-*μ*m membrane and then determined by HPLC/ESI-MS/MS.

### 2.4. HPLC/ESI-MS/MS Analysis

#### 2.4.1. Assay Method

HPLC/ESI-MS/MS analysis was performed on a TSQ Discovery triple-quadrupole mass spectrometer and a surveyor liquid chromatograph (Thermo Fisher Scientific, Waltham, MA, USA). Xcalibur 2.0.7 (Thermo Fisher Scientific) software was used to process the quantitative data obtained from calibration standards and samples.

#### 2.4.2. HPLC Conditions

Methanol combined with 0.1% formic acid and 2 mmol/L ammonium acetate (90 : 10, v/v) in LC-MS grade water were used as mobile phases. The injection volume was 5 *μ*L. The flow rate was set 0.25 mL/min and the column temperature at 30°C, and the autosampler temperature was set at 5°C. The analytical column was Sepax GP-C_18_, 150 mm × 2.0 mm i.d. packed with 3 *μ*m particles (Sepax Technologies, USA).

#### 2.4.3. MS/MS Conditions

**Figure 1:**
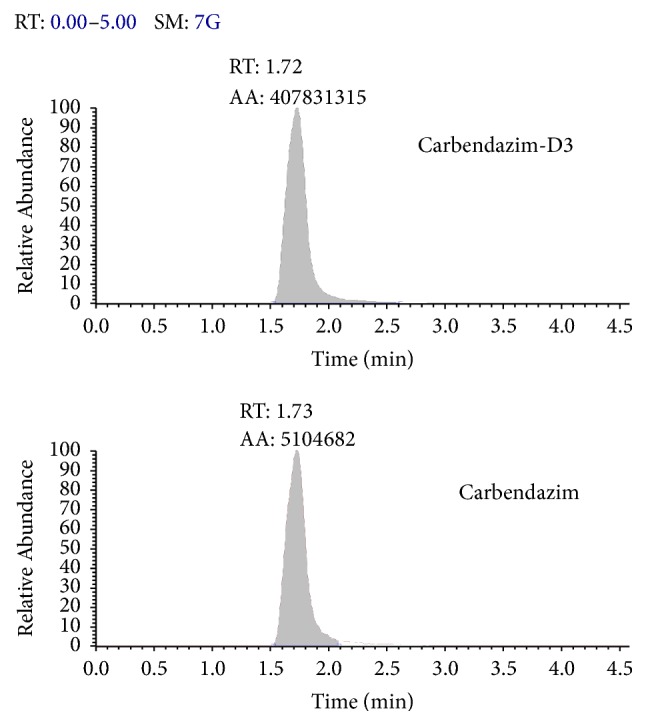
HPLC/ESI-MS/MS chromatograms of carbendazim and carbendazim-D3 in the standard solution.

The electrospray ionization- (ESI-) MS interface was operated in the positive ion mode with selected reaction monitoring (SRM). The ESI source conditions were shown below. Ion spray voltage was 4000 V. Spray needle temperature was set at 350°C. Sheath gas (N_2_) was 35 (arbitrary) units, auxiliary gas (N_2_) was 15 U, and collision gas (Ar) was 1.5 mTorr.  Table 2 showed the MS/MS transitions selected for quantification and confirmation together with the optimized parameters for carbendazim and carbendazim-D3. The retention time of carbendazim and carbendazim-D3 was about 1.73 and 1.72 min in Figure 1.

### 2.5. Transfer Assessment and Mathematical Analysis for Curve Fitting

The transfer assessment of carbendazim residues from rape flowers to apicultural products was figured out by HPLC/ESI-MS/MS method. Mathematical curve fitting was identified by computer associated calculation on the basis of transfer assessment data.

## 3. Results and Discussion

### 3.1. Optimization of Sample Pretreatment

Pollen's pretreatment method developed from a fast and easy multiresidue method employing acetonitrile extraction/partitioning and dispersive solid-phase extraction for the determination of pesticide residues [19]. Honey's pretreatment method was improved from a confirmative method for sulfonamides, trimethoprim, and dapsone in honey by acidic hydrolysis and SPE [20]. Royal jelly's pretreatment method was improved from analysis of tetracycline residues in royal jelly by liquid chromatography-tandem mass spectrometry [21]. All pretreatments were modified and consisted of three steps: firstly, extraction with suitable solvents and, the second step, clean-up by dispersive solid-phase extraction technique. The third step comprised concentration, reconstitution, and filtration. The pretreatments were easy and operative.

### 3.2. Partial Validation of Analysis Method

Partial validation was carried out to evaluate the performances of the method for the quantitative analysis of carbendazim. To set up valid methods, performance characteristics, such as linear equation, linear range, accuracy, precision, limits of detection (LOD), and limits of quantification (LOQ), were determined. The methods were validated partially using the optimized conditions based on analysis time and resolution. The matrix could either enhance or suppress ionization of pesticides during the electrospray process. Therefore, blank samples of apicultural products (pesticide free) obtained from local markets were chosen as reference matrixes. In addition, concentration range of linear equation was chosen according to accuracy and sensitivity of SRM pattern during MS/MS analyses. Working standards at different concentrations were prepared by transferring 0.05, 0.25, 0.5, 2.0, 5.0, and 25.0 mL of 2.0 mg/L intermediate working solution into separate 100 mL volumetric flasks and making up to volume with blank matrix extracts in methanol. Calibration curves were constructed using concentration (mg/L) versus the ratio (carbendazim area/carbendazim-D3 area) in the form of *y* = *ax* + *b*. The precision was the repeatability of signals calculated from 6 dependent injections with lowest concentration within the linear range (*C* = 0.001 mg/L). The accuracy was evaluated by recovery yields with different adding concentrations at the beginning of the processing. LOD was determined as 3 times the signal to noise ratio of the quantitative ion transition by analysis of spiked sample containing carbendazim at low concentration levels with five replicate extractions. LOQ was defined as the lowest spiking level on acceptable recovery.  Table 3 was the partial validation parameters for carbendazim added in different black samples. The results illustrated that the methods were reliable and sensitive to determine carbendazim in apicultural products.

### 3.3. Transfer Assessment

The transfer assessment of carbendazim residues from rape flowers to apicultural products was figured out by HPLC/ESI-MS/MS method.

Concentration of carbendazim in bee pollen was 99.2 ± 20.5 mg/kg 1 day after spraying, while 6.28 ± 0.09 mg/kg 3 days after spraying. That may be due to the rainy weather on 2nd day after spraying. The rain might wash away the pesticide covered on the surface of pollen. The data of 4th day was higher than 3rd day which might be due to uneven application. The residues showed a downward trend and the decline of the speed was more and more slow after 4 days later. The residue on 18th day was at 1.10 ± 0.03 mg/kg.

Concentration of carbendazim in bee honey was highest 6 days after spraying and then reduced. From 6 days to 18 days after spraying, the degradation rate of carbendazim became slower, while it suddenly turned out to be fast from 18 days to 24 days after spraying. The residue on 24th day was at 0.032 ± 0.001 mg/kg.

Concentration of carbendazim in bee jelly was highest 6 days after spraying and then reduced. The degradation rate of carbendazim was very slow after 12 days. The residue on the 22nd day was at 0.077 ± 0.002 mg/kg.

### 3.4. Mathematical Analysis for Curve Fitting

Mathematical analysis for curve fitting was carried out on the basis of transfer assessment of carbendazim residues from rape flowers to apicultural products.

**Figure 2:**
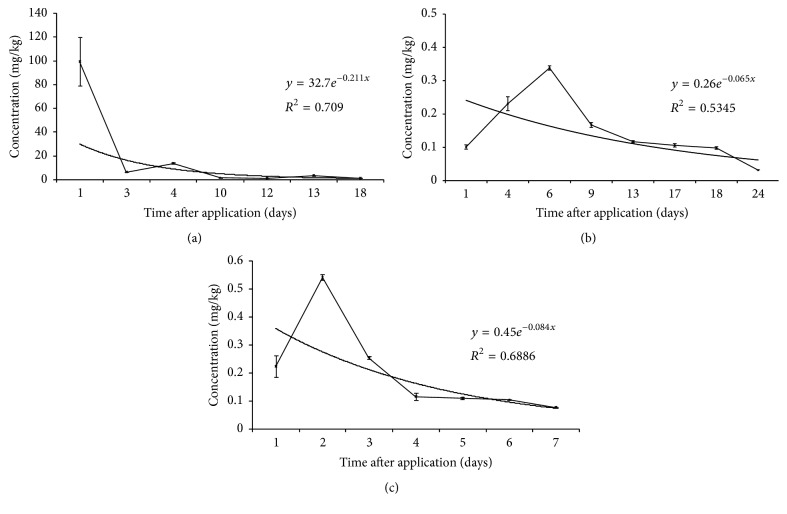
The concentration change of carbendazim in apicultural products established by mathematical analysis for curve fitting (a) pollen; (b) honey; (c) jelly.

The fitting curves were obtained by professional function drawing software including Excel (2013 version) and Origin 9.1. Firstly, *P* value was obtained from Origin 9.1. The curve was fit if *P* < 0.05 [22]. Pesticide residues of corresponding time could be found from the drawn fitting curve [23]. Calibration curves were constructed using time after spraying pesticide application (days) versus carbendazim residues (mg/kg) in the form of exponential function. By mathematical curve fitting, the exponential function simulation of carbendazim residues in pollen was *y* = 32.7*e*^−0.211*x*^ (*R*^2^ = 0.709) as shown in Figure 2(a). *P* value was 4.11*∗*10^−5^ < 0.05; thus the reliability of curve fitting was confirmed. The results obtained from curve fitting showed that the degradation half-life of carbendazim in pollen was about 1.98 days.

In this same way, the exponential function simulation of carbendazim residues in honey was *y* = 0.26*e*^−0.065*x*^ (*R*^2^ = 0.5345) as shown in Figure 2(b). *P* value was 0.0101 < 0.05. According to recommended dose of carbendazim, the results obtained from curve fitting showed that the degradation half-life of carbendazim in honey was about 25.2 days.

By the same method, the exponential function simulation of carbendazim residues in royal jelly was *y* = 0.45*e*^−0.084*x*^ (*R*^2^ = 0.6886) as shown in Figure 2(c). *P* value was 0.0253 < 0.05. The results obtained from curve fitting showed that the degradation half-life of carbendazim in jelly was about 16.7 days.

## 4. Conclusion

The results of this study indicate a practical approach to study pesticide residues from rape flowers to apicultural products by HPLC/ESI-MS/MS method. The method is validated reliable and sensitive to determine carbendazim in apicultural products. Transfer assessment and mathematical curve fitting of carbendazim residues from rape flowers to apicultural products show that the residues of carbendazim are reduced with spraying time. The gap of carbendazim residues between pollen and honey is decreased with time.

The carbendazim residues in pollen are 10 times higher than that in honey and jelly. The study can help farmers to keep safe intervals of corresponding fungicides. The study can provide data support for the analysis of the residue limits and risk assessment of carbendazim in apicultural products and to improve security level of apicultural products.

## Figures and Tables

**Table 1:** Sampling table of field trials.

Sampling date	Weather	Time after application (days)	Greenhouses		
			P_1_ (blank), P_2_~P_4_ (test)		
			Pollen	Honey	Jelly
23 March	Sunny	0			
24 March	Cloudy	1	✓	✓	
26 March	Overcast	3	✓		✓
27 March	Cloudy	4	✓	✓	
29 March	Moderate rain	6		✓	✓
1 April	Cloudy	9		✓	✓
2 April	Overcast	10	✓		
4 April	Cloudy	12	✓		✓
5 April	Sunny	13	✓	✓	
7 April	Rain Shower	15			✓
9 April	Cloudy	17		✓	
10 April	Sunny	18	✓	✓	✓
14 April	Sunny	22			✓
16 April	Rain Shower	24		✓	

**Table 2:** SRM conditions for target compounds.

Compound	Parent mass (m/z)	Product mass (m/z)	Collision energy (V)
Carbendazim	192	132^*∗*^	27
	192	160	17
Carbendazim-D3	195	160^*∗*^	29

^*∗*^Quantitative ion.

**Table 3:** Partial validation parameters for carbendazim added in different blank samples.

Parameters	Pollen	Honey	Jelly
Linear equation	*y* = 2.6182*x* + 0.0016	*y* = 0.8517*x* + 0.0042	*y* = 0.2220*x* + 0.0026
Linear range (mg/L)	0.001–0.5	0.001–0.1	0.001–0.1
*R* ^2^	0.9999	0.9994	0.9998
Repeatability of signals	1.52	1.47	1.36
(RSD, %, *n* = 6 dependent injections and *C* = 0.001 mg/L)			
LOD (mg/kg)	0.001	0.001	0.001
LOQ (mg/kg)	0.01	0.01	0.01
Recover (mean ± RSD), %, (*n* = 6)			
0.01^*∗*^	84.6 ± 7.5	95.7 ± 8.4	102 ± 2.4
0.2^*∗*^	88.3 ± 4.2	101 ± 6.1	116 ± 2.7
1.0^*∗*^	90.2 ± 5.3	110 ± 8.7	100 ± 1.7

^*∗*^(Added  level, mg/kg).
